# EXAMINING THE ASSOCIATION BETWEEN NEIGHBORHOOD CHARACTERISTICS AND TELOMERE LENGTH BY RACE AND URBANICITY

**DOI:** 10.1093/geroni/igz038.2195

**Published:** 2019-11-08

**Authors:** Amy Thierry

**Affiliations:** Xavier University of Louisiana, New Orleans, Louisiana, United States

## Abstract

A dearth of research investigates socio-environmental mechanisms of health disparities. Therefore, this paper tests the association between neighborhood characteristics and telomere length (TL), a biomarker of cellular age, and examines if this relationship differs by race and urban residence. Data from US-born, non-Hispanic Black and White midlife and older adults (n=4,155) in the 2008 Health and Retirement Study was analyzed to test the relationship between TL and perceived neighborhood safety, cleanliness, and social cohesion. Linear regression models stratified by race included interaction terms between neighborhood characteristics and urban residence. Negative perceptions of neighborhood characteristics were associated with shorter TL in Blacks, with urban-dwelling Blacks having shorter TL than Blacks in less urban areas. Findings suggest that negatively perceived neighborhoods may be more detrimental to TL for urban compared to rural Blacks. Future research should elucidate the biobehavioral consequences of socially disadvantaged urban neighborhoods on healthy aging among Black adults.

